# Pomegranate for Your Cardiovascular Health

**DOI:** 10.5041/RMMJ.10113

**Published:** 2013-04-30

**Authors:** Michael Aviram, Mira Rosenblat

**Affiliations:** The Lipid Research Laboratory, The Rappaport Faculty of Medicine and Research Institute, Technion-Institute of Technology, and Rambam Medical Center, Haifa, Israel

**Keywords:** Atherosclerosis, pomegranate, antioxidant, polyphenols, macrophages, lipoproteins (LDL, HDL)

## Abstract

Pomegranate is a source of some very potent antioxidants (tannins, anthocyanins) which are considered to be also potent anti-atherogenic agents. The combination of the above unique various types of pomegranate polyphenols provides a much wider spectrum of action against several types of free radicals. Indeed, pomegranate is superior in comparison to other antioxidants in protecting low-density lipoprotein (LDL, “the bad cholesterol”) and high-density lipoprotein (HDL, “the good cholesterol”) from oxidation, and as a result it attenuates atherosclerosis development and its consequent cardiovascular events. Pomegranate antioxidants are not free, but are attached to the pomegranate sugars, and hence were shown to be beneficial even in diabetic patients. Furthermore, pomegranate antioxidants are unique in their ability to increase the activity of the HDL-associated paraoxonase 1 (PON1), which breaks down harmful oxidized lipids in lipoproteins, in macrophages, and in atherosclerotic plaques. Finally, unique pomegranate antioxidants beneficially decrease blood pressure. All the above beneficial characteristics make the pomegranate a uniquely healthy fruit.

## POMEGRANATE FRUIT COMPOSITION

The pomegranate tree, which is said to have flourished in the Garden of Eden, has been extensively used as a folk medicine in many cultures.^1^ Edible parts of pomegranate fruits (about 50% of the total fruit weight) comprise 80% juice and 20% seeds. Pomegranate juice (PJ) consists of the crushed fruit only. Fresh juice contains 85% moisture, 10% total sugars, 1.5% pectin, and also antioxidants such as ascorbic acid and polyphenols. Content of soluble polyphenols in PJ varies within the limits of 0.2%–1.0%, depending on the variety, and includes mainly anthocyanins (such as cyanidin-3-glycoside, cyanidin-3,3-diglycoside, and delphindin-3-glycoside) and anthoxantins (such as catechins, ellagic tannins, and gallic and ellagic acids). Ellagic acid and hydrolyzable ellagitannins are both implicated in protection against atherogenesis, along with their potent antioxidant capacity. Punicalagin is the major ellagitannin in PJ, and this compound is responsible for the high antioxidant activity of PJ.^2^–^4^

## POMEGRANATE JUICE BIOAVAILABILITY

*In vivo* studies were conducted in order to evaluate whether the active antioxidant components of PJ are absorbed. Recent studies examined the bioavailability and metabolism of punicalagin in the rat as an animal model.^5^,^6^ Two groups of rats were studied. One group was fed for 37 days with standard rat diet (*n* = 5), and the second one with the same diet plus 6% punicalagin (*n* = 5). The daily intake of punicalagin ranged from 0.6 g to 1.2 g. Glucuronides of methyl ether derivatives of ellagic acid and punicalagin were detected in plasma. 6H-Dibenzo [b, d] pyran-6-one derivatives were also observed in the plasma, especially during the last few weeks of the study. In urine, the metabolite urolithin was observed along with 6H-dibenzo [b, d] pyran-6-one derivatives, and they were present as aglycones or as glucuronides. It was concluded that since only 3%–6% of the ingested punicalagin was detected as such, or as metabolites in urine and feces, the majority of this ellagitannin has to be converted to undetectable metabolites or accumulated in non-analyzed tissues. Only traces of punicalagin metabolites were detected in liver or kidney. In humans, following consumption of PJ (180mL) containing 25 mg of ellagic acid and 318 mg of hydrolyzable ellagitannins (as punicalagin), ellagic acid was detected in human plasma 1 hour post-ingestion at a maximum concentration of 32 ng/mL, and by 4 hours it was completely eliminated.^7^

Upon analyzing the influence of the physiological conditions in the stomach and small intestine on pomegranate bioactive compounds bioavailability using an *in vitro* availability method,^8^ it was demonstrated that pomegranate phenolic compounds are available during digestion in a high amount (29%). Nevertheless, due to pH, anthocyanins are largely transformed into non-red forms, or degraded. Thus, active components of PJ are indeed absorbed and subsequently affect biological processes which are related to atherogenesis protection.

## POMEGRANATE CONSUMPTION ATTENUATES ATHEROSCLEROSIS DEVELOPMENT

PJ is suggested as the “heart-healthy” fruit juice,^9^ and it was indeed shown to attenuate cardiovascular diseases.^10^ Measurements of the arterial stiffness of the common carotid arteries in 73 patients with at least one cardiovascular risk factor who consumed PJ (Wonderful variety, 240 mL/day for 1 year), showed trends to increased arterial elasticity in the PJ-treated group versus the placebo-treated group (who received beverage of similar caloric content, flavor, and color).

**Figure 1.**
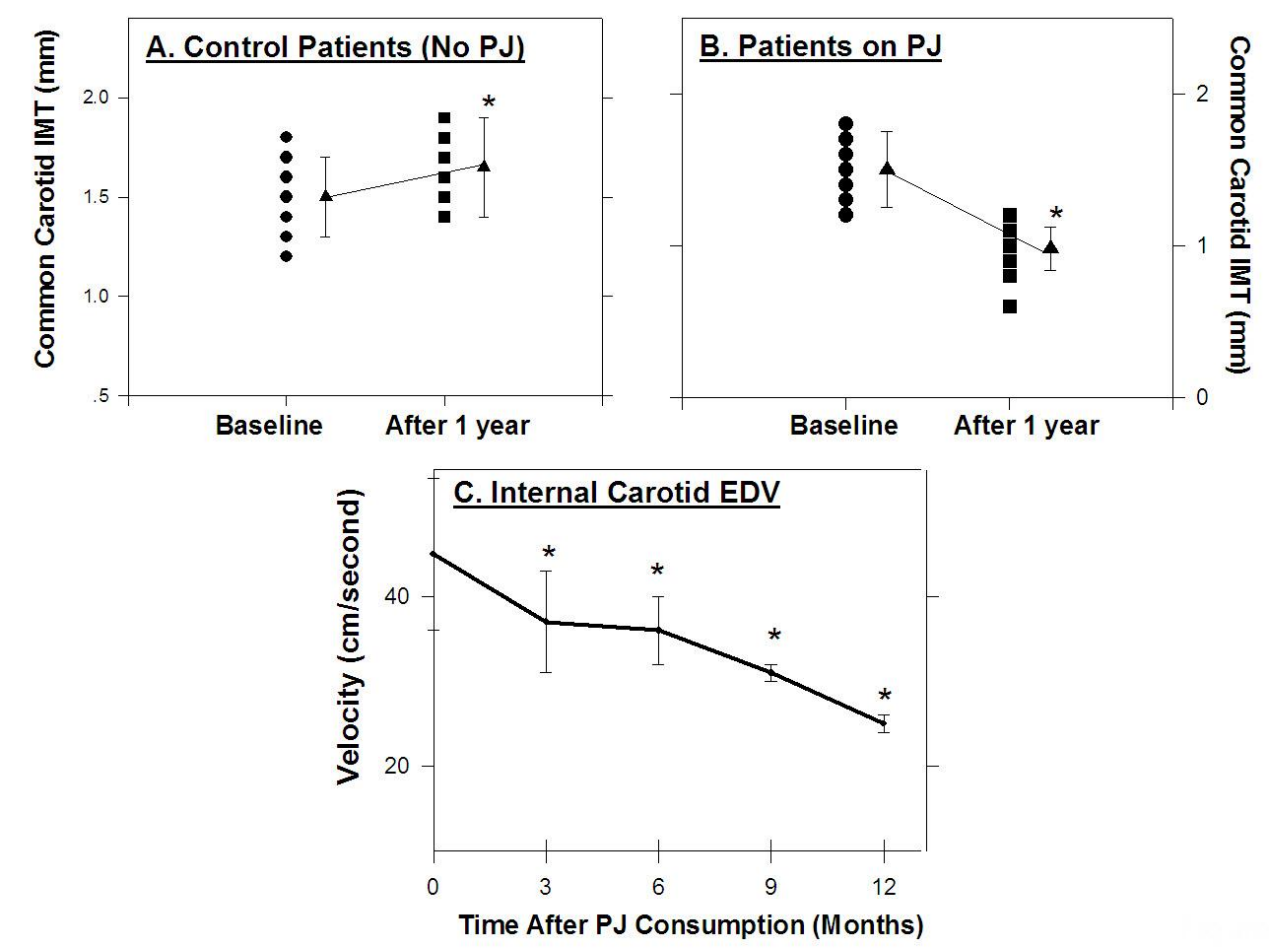
**The effect of PJ consumption by patients with CAS on CIMT and on internal carotid EDV.** Ten patients with CAS were supplemented with PJ for up to 1 year. Common CIMT and EDV were measured in the patients’ left and right carotid arteries before treatment (baseline) and during PJ consumption. The mean values of both carotid arteries are presented. The individual results of mean common carotid artery IMT, as well as the averages of all mean values ±SEM, are shown (A and B). The averages of all mean values ±SEM are shown for internal carotid artery EDV (C). *P<0.01 (after PJ consumption versus Baseline—“0” time).

The effect of a daily consumption of PJ for 3 months on myocardial perfusion in 45 patients who had coronary heart disease (CHD) was also studied.^11^ Patients were randomly assigned into one of two groups: a PJ group (240 mL/day) or a placebo group. The experimental and control groups showed similar levels of stress-induced ischemia at baseline. After 3 months, however, the extent of stress-induced ischemia decreased in the pomegranate group, but increased in the control group. This benefit was observed without changes in cardiac medications, blood sugar, hemoglobin A1c, body weight, or blood pressure, in either group.^11^ We next investigated the effects of PJ consumption by patients with carotid artery stenosis (CAS) on carotid lesion size, in association with changes in oxidative stress.^12^ Ten patients were supplemented with PJ for up to 1 year, and nine CAS patients who did not consume PJ served as a control group. Blood samples were collected before treatment and after 3, 6, 9, and 12 months of PJ consumption. Patients’ carotid intima-media thickness (CIMT) was compared between the PJ group and the control group. While in the control patients group (no PJ) CIMT increased by 10% after 1 year (Figure 1A), PJ consumption resulted in a significant CIMT reduction, by up to 35% (Figure 1B). Analysis of the mean CIMT (of the left and right common carotid arteries) before and during PJ consumption revealed a gradual reduction of 13%, 22%, 26%, and 35%, as observed after 3, 6, 9, and 12 months of PJ consumption, respectively, in comparison to baseline values. On examination of the internal carotid arteries, flow velocities were calculated at the stenotic sites and expressed by peak systolic velocity (PSV) and end-diastolic velocity (EDV). The ultrasound outcome data were the change over time in maximal IMT, which was measured in the same preselected carotid artery segments. Twelve months of PJ consumption resulted in PSV reduction by 12% and 28% in the left and the right carotid arteries, respectively. Mean carotid EDV of both left and right carotid arteries gradually decreased, by 16%, 20%, 31%, and 44% after 3, 6, 9, and 12 months of PJ consumption, respectively (Figure 1C).^12^

A randomized, double-blind trial assessed the influence of PJ consumption on anterior and posterior CIMT progression rates in subjects at moderate risk for coronary heart disease. Subjects were men (45–74 years old) and women (55–74 years old) with one or more major CHD risk factors and baseline posterior wall CIMT of 0.7–2.0 mm, without any significant stenosis. Participants consumed 240 mL/day of PJ (*n* = 146), or a control beverage (*n* = 143) for up to 18 months. No significant difference in overall CIMT progression rate was observed between PJ and control treatments. In exploratory analyses, however, of subjects in the most adverse tertiles for baseline serum lipid peroxides, triglycerides (TGs), high-density lipoprotein (HDL) cholesterol, TGs/HDL cholesterol, total cholesterol/HDL cholesterol, and apolipoprotein-B100, those in the PJ group had significantly less anterior wall and/or composite CIMT progression versus control subjects. These results suggest that, in subjects at moderate CHD risk, PJ consumption had no significant effect on overall CIMT progression rate, but slowed CIMT progression in subjects with increased oxidative stress and disturbances in the TG-rich lipoprotein/HDL axis.^13^

## INHIBITORY EFFECT OF POMEGRANATE CONSUMPTION ON SERUM LIPID PEROXIDATION

The oxidative modification hypothesis of atherosclerosis proposes that low-density lipoprotein (LDL) oxidation plays a pivotal role in early atherogenesis. This hypothesis is supported by evidence that oxidized LDL (Ox-LDL) is present in atherosclerotic lesions, and in human plasma from patients with cardiovascular diseases, and it correlates with the presence of angiographically documented complicated plaques,^14^–^17^ thus identifying those patients who are at increased risk for future myocardial infarction (MI), independently of other risks. Since PJ contains very potent antioxidants, it can attenuate atherosclerosis development by reducing oxidative stress in these patients. Indeed, human plasma obtained from healthy subjects after 2 weeks of PJ consumption (50mL PJ concentrate/day, equivalent to 1.5 mmol total polyphenols) demonstrated a small but significant (*P*<0.01) 16% decreased susceptibility to free radical-induced lipid peroxidation, in comparison to plasma obtained prior to PJ consumption, as measured by lipid peroxides formation, or by total antioxidant status (TAS) in serum.^18^ To determine the effect of increasing or decreasing the dosages of PJ on plasma lipid peroxidation, and to analyze the capability of PJ to maintain its effect after termination of juice consumption, three subjects were further studied. Supplementation of 20 mL of PJ concentrate/day for one week resulted in a significant decrease of 11% in plasma lipid peroxidation, compared to plasma obtained prior to PJ consumption. Supplementation of 50 mL PJ concentrate/day for one more week exhibited a further 21% decrease in plasma lipid peroxidation. However, a further increase in the supplemented PJ to 80 mL of PJ concentrate/day for an additional one week did not further inhibit plasma susceptibility to lipid peroxidation. Gradual decreasing of the PJ dosage in these three subjects down to 40 mL/day for one week, and then to 20 mL/day for an additional two weeks, did not significantly affect plasma lipid peroxidation, which remained low in comparison to the levels obtained after supplementation of 80 mL of PJ concentrate/day. Two weeks after cessation of PJ supplementation the reduced rate of plasma susceptibility to lipid peroxidation was sustained. After a further four weeks with no PJ consumption, plasma lipid peroxidation returned to the higher values obtained before PJ consumption.^18^

**Figure 2.**
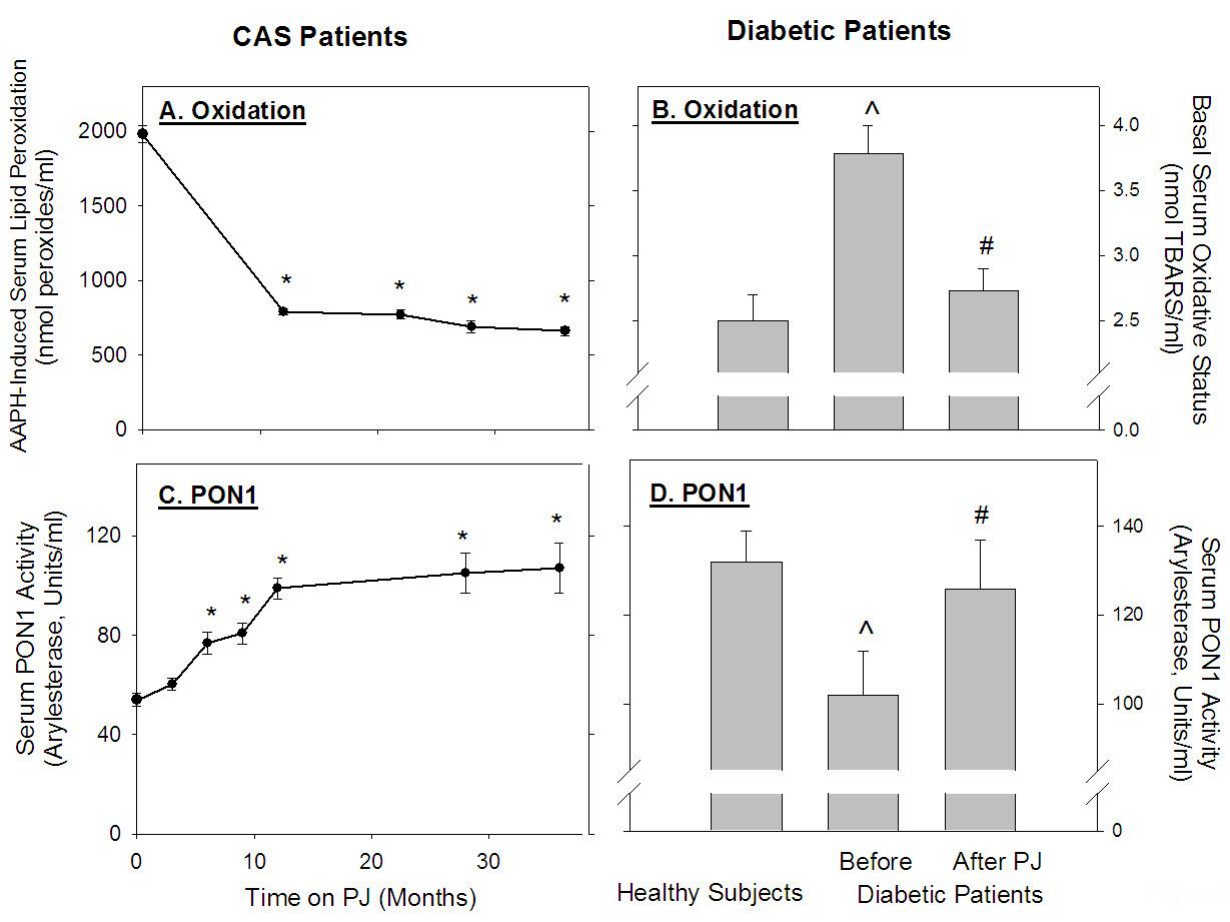
**The effect of PJ consumption by patients with CAS, or by diabetic patients, on their serum oxidative stress and on serum PON1 activity.** **A and C:** Ten patients with CAS were supplemented with PJ for a 1-year period. Blood samples were drawn from the patients before, and after 3, 6, 9, and 12 months of PJ consumption. **B and D:** Ten patients with type 2 diabetes mellitus were supplemented with PJ for 3 months. Blood samples were drawn from the patients before, and after PJ consumption and also from 10 healthy subjects. **A:** The serum susceptibility to oxidation by 2,2′-azobis amidinopropane hydrochloride (AAPH) was determined by the lipid peroxides assay. **B:** Basal serum oxidative status was determined by the level of thiobarbituric acid reactive substances (TBARS). **C and D:** Serum PON1 arylesterase activity was measured using phenyl acetate as the substrate. Results are expressed as mean ±SD (*n*=10). *P<0.01 (after PJ consumption versus before treatment), ^*P*<0.01 (diabetic patients before versus healthy subjects), #*P*<0.01 (diabetic patients after PJ versus before).

The effect of PJ consumption by patients with CAS on their serum oxidative state was also measured.^12^ A significant (*P*<0.01) reduction in the concentration of antibodies against Ox-LDL by 24% and 19% was observed after 1 and 3 months of PJ consumption, respectively (from 2070±61 EU/mL before treatment to 1563±69 and 1670±52 EU/mL after 1 and 3 months of PJ consumption, respectively). TAS in serum from these patients was substantially increased 2.3-fold (from 0.95±0.12 nmol/L at baseline, up to 2.20±0.25 nmol/L after 12 months of PJ consumption). These results indicate that PJ administration to patients with CAS substantially reduced their serum oxidative status and could thus inhibit plasma lipid peroxidation. The susceptibility of the patients’ serum to free radical-induced oxidation decreased after 12 months of PJ consumption by up to 62% (Figure 2A). Increased oxidative stress was observed in the serum of non-insulin-dependent type 2 diabetes mellitus patients versus healthy subjects (Figure 2B). Consumption of 50mL of PJ per day for a period of 3 months resulted in a significant reduction in the basal serum thiobarbituric acid reactive substances (TBARS) levels, by 28% (Figure 2B).^19^ Consumption of PJ for 1 and 2 weeks by healthy volunteers increased the resistance of their LDL to copper ion-induced oxidation, as shown by a prolongation of the lag time required for the initiation of LDL oxidation, by 29% and 43%, in comparison to LDL obtained prior to juice consumption. Similarly, the resistance of their HDL to copper ion-induced oxidation also gradually increased after PJ consumption, as shown by a prolongation in the lag time required for the initiation of HDL oxidation from 37±2 minutes to 45±6 minutes before and 2 weeks after PJ consumption, respectively.^18^ PJ consumption by patients with CAS resulted in a significant reduction in the basal level of LDL-associated lipid peroxides by 43%, 89%, 86%, and 90% after 3, 6, 9, and 12 months of PJ consumption, respectively, and, in parallel, it increased the resistance of LDL to copper ion-induced oxidation. This was demonstrated by reduced formation of lipid peroxides in LDL during its incubation with copperions (by 40%, 49%, 57%, and 59% after 3, 6, 9, and 12 months of PJ consumption, respectively).^12^

## THE STIMULATORY EFFECT OF POMEGRANATE CONSUMPTION ON SERUM PARAOXONASE 1 (PON1)

Most of the serum antioxidant and anti-atherogenic enzyme, PON1, is HDL-associated.^20^ Still, low levels of PON1 are also associated with chylomicrons and with very-low-density lipoprotein (VLDL), but not with LDL.^21^ PON1 has a protective role in the attenuation of cardiovascular diseases.^22^ Serum PON1 concentration and activity are better predictors of the risk for cardiovascular diseases than the PON1 genotype.^23^ A negative association was observed between serum PON1 activity and IMT in subjects with CHD.^24^ Attenuation of atherosclerosis by PON1 can result from its ability to hydrolyze specific oxidized lipids in lipoproteins,^25^ in arterial wall cells (including macrophages),^26^,^27^ and in atherosclerotic lesions.^28^

The increased resistance of LDL and of HDL to oxidation after PJ administration to healthy subjects, or to patients with CAS, could have also resulted from increased serum HDL-associated PON1 activity. Indeed, a significant 18% increase in serum PON1 activity was monitored in healthy subjects after PJ consumption for a period of 2 weeks.^18^ In CAS patients, serum PON1 arylesterase activity significantly increased by 11%, 42%, 49%, and 83% after 3, 6, 9, and 12 months of PJ consumption, respectively (Figure 2C),^12^ and in patients with type 2 diabetes mellitus it significantly increased by 12% after PJ consumption for 3 months (Figure 2D).^19^

**Figure 3.**
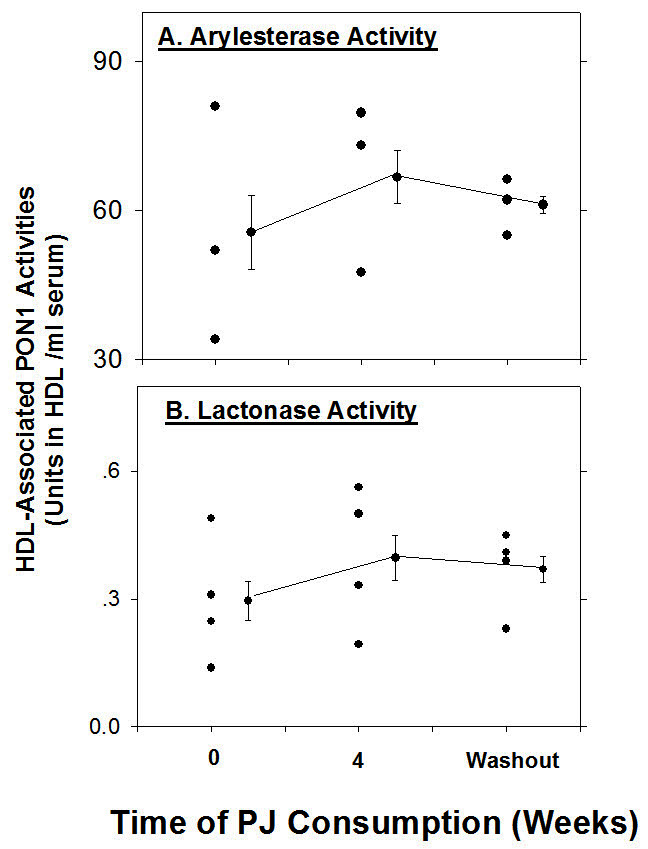
**The effect of PJ consumption by diabetic males on their HDL-associated PON1 activities.** Ten male patients with type 2 diabetes mellitus consumed 50 mL of concentrated pomegranate juice (PJ) per day (which contain 1.5 mmol of total polyphenols) for a period of 1 month, followed by a period of 4 weeks’ “wash-out.” Blood samples were collected from the patients before (0 time) and 4 weeks after PJ consumption, as well as after the “wash-out” period. The HDL fractions were isolated from the blood samples of four patients by ultracentrifugation. The HDL-associated PON1 arylesterase (A), and lactonase activities (B) were determined. The individual results, as well as the mean±SD are shown.

The increment in PON1 protein could result from the direct effect of PJ on PON1 expression in the liver.^29^ The PJ-induced increment in PON1 activity could also result from the reduction in oxidative stress, since oxidized lipids inactivate PON1.^30^ In addition, association of PON1 with HDL stabilizes the enzyme and stimulates its lactonase activity.^20^ In diabetic patients, PON1 dissociates from HDL, and as a consequence, it is less biologically active.^31^ We thus investigated the effects of PJ and POMxl (an extract of the pomegranate outer peel) consumption on PON1 association with HDL in diabetic patients.^32^ HDL-associated PON1 arylesterase and lactonase activities increased significantly after PJ consumption, by 34%–45%, as compared to the baseline levels (Figure 3). In male patients who consumed POMxl, and in female patients who consumed PJ, a similar pattern was observed, although to a lesser extent. PON1 protein binding to HDL was significantly increased by 32% following PJ consumption, while the level of PON1 in the lipoprotein-deficient serum (LPDS) decreased by 62%, suggesting that PJ consumption resulted in increased free PON1 binding to the HDL. A similar trend of increased PON1 protein association with HDL was observed in males following POMxl consumption, as after 4 weeks of POMxl consumption HDL-bound PON1 protein increased by 17% compared to baseline values. The above results were confirmed also in *in vitro* study where serum from diabetic patients was incubated with PJ or with punicalagin, or with no addition (control), for 2 hours at 37°C. Then, HDL was isolated from the serum by ultracentrifugation, and Western blot analysis was performed. After serum incubation with PJ (18μg gallic acid equivalents (GAE)/mL) or with punicalagin, the protein content of HDL-bound PON1 significantly increased by 36% and by 14%, respectively, as compared to control serum. Upon increasing the concentration of PJ or punicalagin up to 36μg GAE/mL, HDL-bound PON1 protein further increased, and it was 62% or 83% higher than that observed in control patients’ serum (no PJ), respectively.

We thus conclude that PJ as well as POMxl consumption by diabetic patients contributes to PON1 stabilization by increasing its association with HDL and therefore enhancing PON1 catalytic activities. The ratio between HDL-associated PON1 and free PON1 gradually decreased as the extent of HDL oxidation increased. The antioxidants vitamin E or PJ inhibited the oxidation-mediated redistribution of PON1 in serum. Indeed, PJ and its purified major polyphenols punicalagin, gallic acid, and ellagic acid all increased PON1 binding also to HDL.^33^ Furthermore, PON1 associated more efficiently with HDLs isolated from diabetic patients after PJ consumption versus the patients’ HDL isolated prior to PJ consumption.^33^

## THE INHIBITORY EFFECT OF POMEGRANATE CONSUMPTION ON BLOOD PRESSURE

As some antioxidants were recently shown to reduce blood pressure (BP), we studied the effect of PJ consumption (50mL, 1.5mmol of total polyphenols per day, for 2 weeks) by hypertensive patients on their BP and on serum angiotensin-converting enzyme (ACE) activity.^34^ A 36% decrement in serum ACE activity and a 5% reduction in systolic BP were noted. A similar dose-dependent inhibitory effect (31%) of PJ on serum ACE activity was observed also *in vitro*. As reduction in serum ACE activity, even with no decrement in blood pressure BP, was previously shown to attenuate atherosclerosis, PJ can offer a wide protection against cardiovascular diseases which could be related to its inhibitory effect on oxidative stress and on serum ACE activity.

In CAS patients the systolic BP was significantly (*P*<0.05) reduced by 7%, 11%,10%, 10%, and 12% after 1, 3, 6, 9, and 12 months of PJ consumption, respectively, compared to values obtained before treatment. In contrast, PJ consumption had no significant effect on the patient’s diastolic blood pressure.^12^

In another study, healthy participants consumed 330mL/day of PJ or control drink for 4 weeks.^35^ Measurements were made at baseline and at 4 weeks. There was a significant fall in systolic BP (−3.14 mmHg, *P* < 0.001), diastolic BP(−2.33 mmHg, *P* < 0.001), and mean arterial pressure (−2.60 mmHg, *P* < 0.001). The fall in BP was not paralleled by changes in concentration of serum ACE.

The effect of PJ supplementation for a short term was also analyzed.^36^ Nineteen young, healthy men completed a randomized, controlled cross-over trial. The active drink (containing a pomegranate extract) was consumed during a high-fat meal (ET-DUR) or 15 min before (ET-PRE), and the placebo drink (no pomegranate extract) was consumed during the high-fat meal (control). Postprandial lipemia was assessed by venous plasma triglyceride concentration. Blood pressure and digital volume pulse, to measure reflection index (DVP-RI) and stiffness index (DVP-SI), were monitored at baseline and at 2 and 4 hours. Systolic BP increased in the ET-PRE and ET-DUR groups to a lesser extent than in the control group (treatment effect *P* = 0.041). There were no treatment effects for DVP-RI, DVP-SI, or diastolic BP. In conclusion, consumption of a single drink containing ellagitannin-rich pomegranate extract did not decrease postprandial plasma triglyceride concentrations, but suppressed the postprandial increase in systolic BP following the high-fat meal.^36^ More clinical research is needed as a number of the studies discussed include small sample sizes and few studies seem to have been undertaken in the recent 5–10 years.^37^

## THE INHIBITORY EFFECT OF POMEGRANATE CONSUMPTION ON MACROPHAGE ATHEROGENICITY

**Figure 4.**
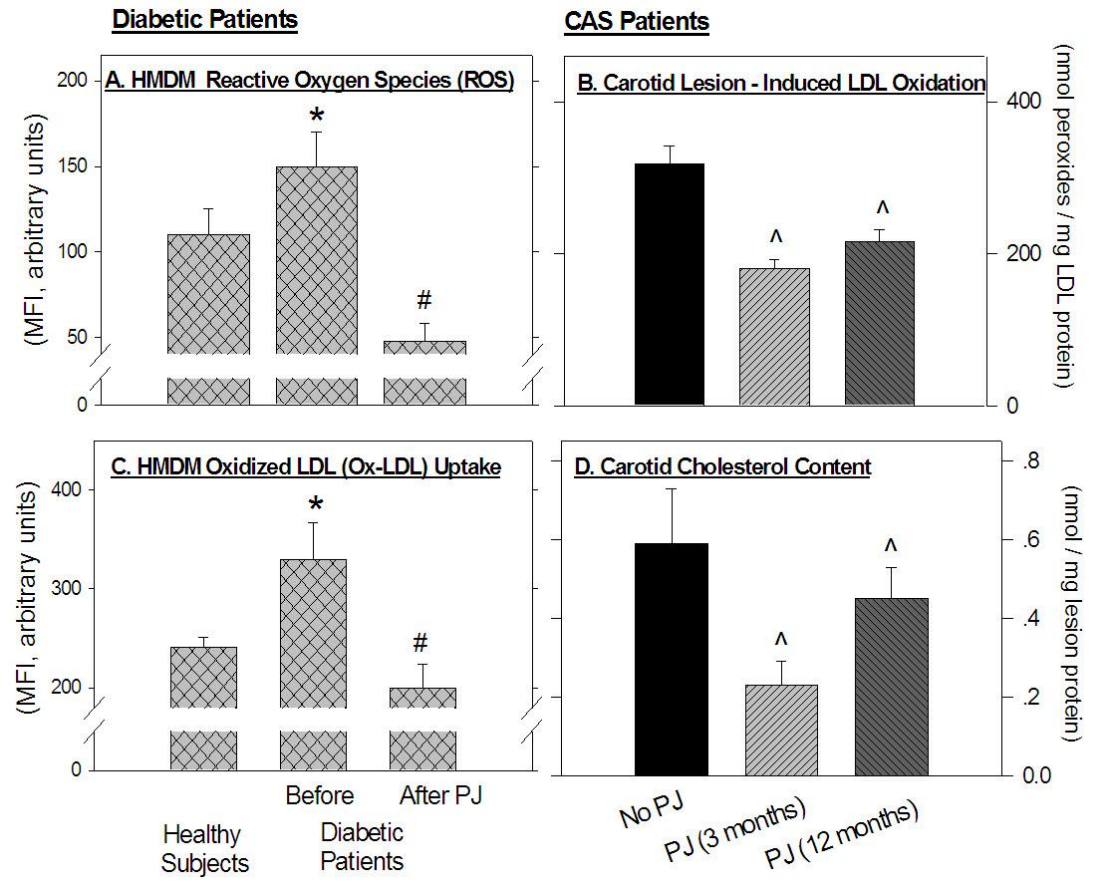
**The anti-atherogenic effects of PJ consumption on HMDM from diabetic patients, and on carotid lesions from patients with CAS.** **A and C:** Monocytes were isolated from the blood of two healthy subjects and from three patients with type 2 diabetes before and after 3 months of PJ consumption (50mL per day). The monocytes were differentiated into macrophages in the presence of RPMI medium containing 10% autologous serum. After 7 days in culture the amount of ROS (**A**) and the uptake of Ox-LDL (20μg of protein/mL) labeled with FITC by the cells (**C**) were determined. Results are given as mean ± SEM.*P<0.01 diabetic patients’ HMDM versus healthy subjects’ HMDM, ^#^*P*<0.01 diabetic patients’ HMDM after PJ consumption versus diabetic patients’ HMDM before PJ consumption. **B and D:** Lesions were collected from seven patients with CAS after endartherectomy and from two patients who consumed PJ for 3 and 12 months and had to undergo endartherectomy during the study, due to clinical deterioration. **B:** Lesions (0.3 g) were incubated with LDL (100 mg of protein/L) in PBS for 20 hours at 37°C. The extent of LDL oxidation was measured by the lipid peroxide assay. Lesion-mediated oxidation of LDL was calculated by subtracting the values obtained in control LDL (incubation with no lesion) from those obtained after LDL incubation with the lesions. Three determinations were done on each lesion. **D:** The amount of cholesterol was measured in the lesion homogenates. Results represent mean ± SEM. ^*P*<0.01 (carotid lesions after PJ consumption versus carotid lesions, no PJ).

Macrophage cholesterol, triglyceride, and oxidized lipids accumulation and foam cell formation are the hallmarks of early atherogenesis.^38^–^40^ Cholesterol accumulation in macrophages can result from impaired balance between external and internal cholesterol sources. LDL is oxidized *in vivo* by arterial wall cells.^41^,^42^ Ox-LDL is taken up by macrophages at an enhanced rate via scavenger receptors which, unlike the LDL receptor, are not down-regulated by intracellular cholesterol content and therefore lead to accumulation of cholesterol in the cells. The cellular cholesterol levels are determined also by the cholesterol biosynthesis rate and by the rate of HDL-mediated cholesterol efflux. We have demonstrated increased oxidative stress in human monocyte-derived macrophages (HMDM) isolated from patients with type 2 diabetes mellitus versus healthy subjects (Figure 4A). After consumption of PJ for 3 months, the patients’ HMDM produced less reactive oxygen species (ROS) in comparison to HMDM before PJ consumption (Figure 4A), respectively. Similarly, incubation of the human carotid lesions from CAS patients with LDL (100μg of protein/mL) for 18 hours under oxidative stress (in the presence of copper ions), revealed that PJ consumption for 3 or 12 months resulted in 43% and 32% reduced capacity of the lesion to oxidize LDL, respectively (Figure 4B). The reduction in cellular oxidative stress can result from PJ-induced increment in PON1, and/or from PJ-induced increment in paraoxonase 2 (PON2).^43^ PON2 is expressed in arterial wall cells including macrophages,^44^ and it protects the cells from oxidative stress and apoptosis.^45^

PJ consumption by patients with type 2 diabetes mellitus significantly decreased the extent of Ox-LDL cellular uptake by their HMDM (by 36%, Figure 4C), as was shown *in vitro* in J774A.1 macrophages.^46^

The carotid lesions from CAS patients who consumed PJ contained also less cholesterol (Figure 4D). This could be related to the reduction in the amount of Ox-LDL and thus in Ox-LDL uptake, and also to PJ-induced increment in PON1, since PON1 was shown *in vitro* to inhibit macrophage cholesterol biosynthesis.^47^ In addition, PJ can directly attenuate cholesterol biosynthesis by the cells, as was previously shown.^46^ The reduction in lesion cholesterol levels after PJ consumption could also result from stimulation of HDL-mediated cholesterol efflux by PON1.^48^

## ANTI-ATHEROGENICITY OF STATIN TREATMENT IN COMBINATION WITH POMEGRANATE

Statins therapy made a significant health benefit, mainly in cardiovascular protection,^49^ by improving the symptoms of atherosclerosis development.^50^ Statins are potent inhibitors of HMGCoA-reductase (the rate-limiting enzyme in cholesterol biosynthesis^51^), and they possess minor antioxidative properties.^52^ However, statins have also deleterious side-effects when taken at high dosages for a long period of time.^53^

Phytosterols, which encompass plant sterols and stanols, are steroid compounds similar in their structure to cholesterol.^54^ The richest sources of phytosterols are vegetable oils and products made from them. The most commonly occurring phytosterols in human diet are β-sitosterol, campesterol, and stigmasterol.^54^ Phytosterols consumption decreased serum cholesterol levels in dyslipidemic patients, as well as their cardiovascular risk.^55^,^56^ Thus, phytosterols were suggested as an appropriate additional therapy to a low-dosage statin treatment.

We have recently^57^ analyzed *in vitro* the anti-atherogenic effects on macrophage cholesterol biosynthesis rate, and on cellular oxidative stress, of the combination of simvastatin with punicalagin, or with a phytosterol (β-sitosterol), or with PJ (that contains both of them^58^,^59^). Simvastatin (15μg/mL) decreased the J774A.1 macrophage cholesterol biosynthesis rate by 42% as compared to control cells. The addition to the statin of either punicalagin (15 or 30µM) or β-sitosterol (50 or 100µM) increased the inhibitory effect of the statin up to 62% or 57%, respectively. Similarly, the combination of PJ and simvastatin resulted in an inhibitory effect up to 59%. While simvastatin inhibited the enzyme HMGCoA-reductase, punicalagin, β-sitosterol, and PJ inhibited macrophage cholesterol biosynthesis downstream to mevalonate. Simvastatin (15μg/mL) also modestly decreased macrophage ROS formation by 11%. In the presence of punicalagin (15 or 30μM), however, a remarkable further inhibition was noted (by 61% or 79%, respectively).Although β-sitosterol alone showed some pro-oxidant activity, the combination of simvastatin, β-sitosterol, and punicalagin clearly demonstrated a remarkable 73% reduction in ROS production. Similarly, simvastatin + PJ decreased the extent of ROS formation by up to 63%.

These results suggest that PJ consumption by hypercholesterolemic patients together with treatment with a low dose of statins could lead to attenuation of macrophage foam cell formation and atherogenesis in these patients.

## CONCLUSIONS

Pomegranate fruit polyphenols protect against lipid peroxidation in serum by direct interaction of pomegranate polyphenols with LDL, or indirectly by increasing serum PON1 stability (HDL association), as well as its catalytic activities, resulting in the hydrolysis of lipid peroxides. Moreover, PJ has a remarkable effect on macrophage and lesion atherogenicity. Pomegranate juice consumption decreased oxidative stress in macrophages and in atherosclerotic lesions, and the extent of Ox-LDL uptake by macrophages. This could be a direct antioxidant effect of PJ, or an indirect effect, by increasing HDL-associated PON1 as well as cellular PON2. Interestingly, the lesion cholesterol levels were decreased after PJ consumption. This could be related to the reduction in Ox-LDL uptake by macrophages, to PJ/PON1-induced inhibition of cholesterol biosynthesis, and to PON1 stimulation of HDL-mediated cholesterol efflux from arterial macrophages.

All these antioxidative and anti-atherogenic effects of pomegranate polyphenols were clearly demonstrated *in vivo*, in humans (healthy subjects, CAS patients, as well as diabetic patients).The preferred pomegranate product in terms of biological potency and consequent health benefits is PJ from the whole fruit (including the peel). Since the combination of antioxidants that exists in PJ can provide a wider range of free radical scavenging capacities than an individual antioxidant, clinical and nutritional studies in humans should be directed towards the use of combinations of several types of dietary antioxidants, as well as combinations of flavonoids together with other nutritional antioxidants, such as vitamin E or carotenoids. In addition, PJ can be beneficially used in combination with low-dose statins in hypercholesterolemic patients.

Finally, it is also important to identify populations suitable for antioxidant treatment, as antioxidants treatment may be beneficial only in subjects who are under excess oxidative stress.

## Abbreviations:

AAPH: 2,2′-azobis amidinopropane hydrochloride;
ACE: angiotensin-converting enzyme;
BP: blood pressure;
CAS: carotid artery stenosis;
CHD: coronary heart disease;
CIMT: carotid intima-media thickness;
EDV: end-diastolic velocity;
GAE: gallic acid equivalents;
HDL: high-density lipoprotein;
HMDM: human monocyte-derived macrophages;
LDL: low-density lipoprotein;
LPDS: lipoprotein-deficient serum;
MI: myocardial infarction;
Ox-LDL: oxidized LDL;
PJ: pomegranate juice;
POMxl: an extract of the pomegranate outer peel;
PON: paraoxonase;
PSV: peak systolic velocity;
ROS: reactive oxygen species;
TAS: total antioxidant status;
TBARS: thiobarbituric acid reactive substances;
TGs: triglycerides;
VLDL: very-low-density lipoprotein.
